# Correction: LC-MS/MS characterization of pirtobrutinib impurities and product degradation: stability studies

**DOI:** 10.1039/d5ra90116b

**Published:** 2025-10-20

**Authors:** Modachakanahally K. Pavithra, Chaya G., Hemavathi N. Deepakumari, Hosakere D. Revanasiddappa, Salah Jasim Mohammed, Hasan Sh. Majdi, Abdullah H. Alsabhan, Shareefraza J. Ukkund

**Affiliations:** a Department of Chemistry, Bharathi College Bharathinagara Mandya 571 422 Karnataka India pavithramk664@gmail.com chayaguruswamy97@gmail.com; b Department of Chemistry, Regional Institute of Education (NCERT) Bhubaneswar 751022 Odisha India deepakumari_22@yahoo.com; c Department of Chemistry, University of Mysore Manasagangotri Mysuru 570 006 Karnataka India hdrevanasiddappa@yahoo.com; d Civil Engineering Department, Dijlah University College Baghdad 00964 Iraq salah.jasmin@duc.edu.iq; e Department of Chemical Engineering and Petroleum Industries, Al-Mustaqbal University College Babylon 51001 Iraq mn13022020@gmail.com; f Department of Civil Engineering, College of Engineering, King Saud University Riyadh 11421 Saudi Arabia aalsabhan@ksu.edu.sa; g Department of Biotechnology, P.A. College of Engineering Mangalore 574153 India drshareef2022@gmail.com

## Abstract

Correction for ‘LC-MS/MS characterization of pirtobrutinib impurities and product degradation: stability studies’ by Modachakanahally K. Pavithra *et al.*, *RSC Adv.*, 2024, **14**, 34868–34882, https://doi.org/10.1039/D4RA06299J.

The authors regret an error in Table 4 in the original article. In the original article, the data values for ‘Amount found (mg)’ and ‘% Recovery’ were reported incorrectly. A corrected Table 4 is displayed below.

**Table 4:** Accuracy results of pirtobrutinib (*n* = 5) through HPLC technique

Concentration (at specification level)	Area	Amount added (mg)	Amount found (mg)	% Recovery	Mean % recovery
50%	1 684 579	50.00	50.3	100.6	100.8
	1 679 845	50.00	50.2	100.4	
	1 695 483	50.00	50.7	101.4	
100%	3 348 458	100.00	100.1	100.1	100.3
	3 357 376	100.00	100.4	100.4	
	3 347 302	100.00	100.3	100.3	
150%	5 019 771	150.00	150.4	100.3	100.9
	5 092 322	150.00	152.1	101.4	
	5 066 740	150.00	151.8	101.2	

The authors sincerely apologise for this oversight. The conclusions of this article are not affected by these changes.

An independent expert has reviewed the corrected information and deemed that a Correction is appropriate in this case.

The Royal Society of Chemistry apologises for these errors and any consequent inconvenience to authors and readers.

